# Supplementary material for: A near complete genome assembly of chia assists in identification of key fatty acid desaturases in developing seeds
Source: Front Plant Sci. 2023 Mar 20;14:1102715. doi: 10.3389/fpls.2023.1102715 (PMC10067618; doi:10.3389/fpls.2023.1102715)
Supplement: Supplementary file 5 [file DataSheet_1.pdf]

Supplemental Table 1. Basic statistics of the sequencing data that used for assemble the chia genome.

Data type	Sequencing mode	Number of reads	Bases
HiFi	PacBio circular consensus sequencing	1,532,222	24,679,796,137
Genome survey	Illumina paired-end 150 bp	60,425,916	9,063,887,400
Hi-C	Illumina paired-end 150 bp	424,922,686	63,738,402,900

Supplemental Table 6. Basic statistics of results from three genome assemblers.

Genome assembler	Number of contigs	Total length	Max length	Min length	N50
Hifiasm-0.16.1-r375	666	388,048,784	49,694,750	6,366	21,830,104
HiCanu-2.2	2,495	476,070,943	33,199,228	8,611	12,791,107
Flye-2.9-b1768	923	389,350,020	12,983,611	504	1,762,795

A

B

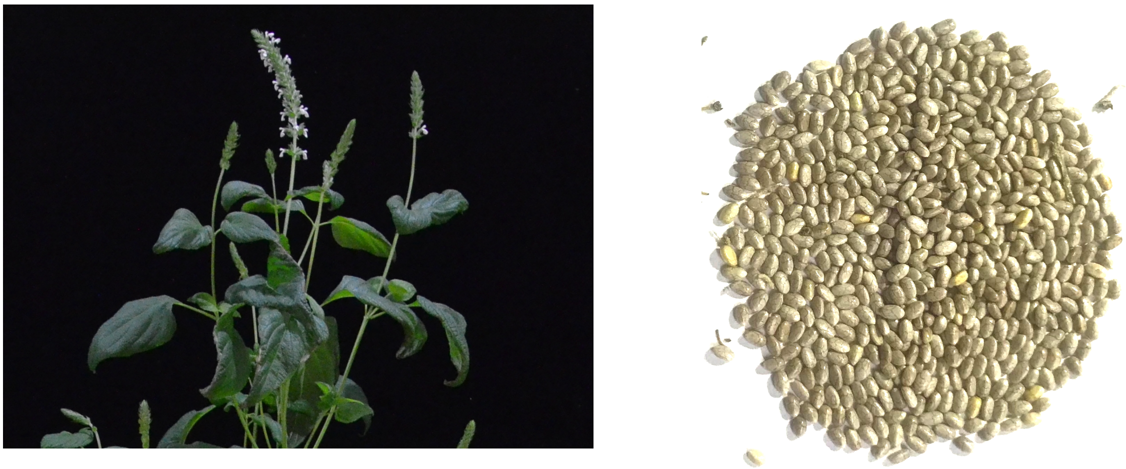

Supplemental Figure 1. Chia plant (**A**) and seeds (**B**) used for genome assembly.

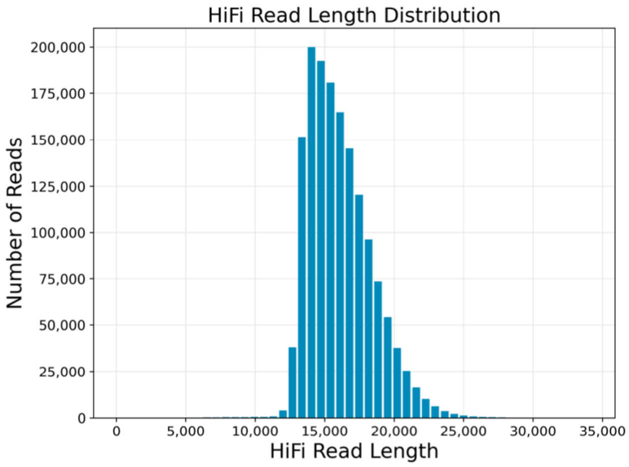

Supplemental Figure 2. The length distribution of HiFi reads.

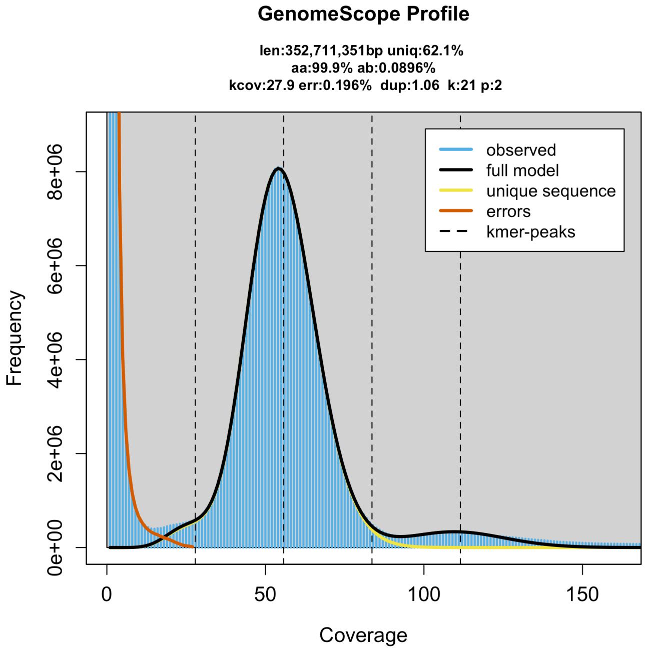

Supplemental Figure 3. Observed and modeled k-mer distribution of HiFi reads using GenomeScope2.

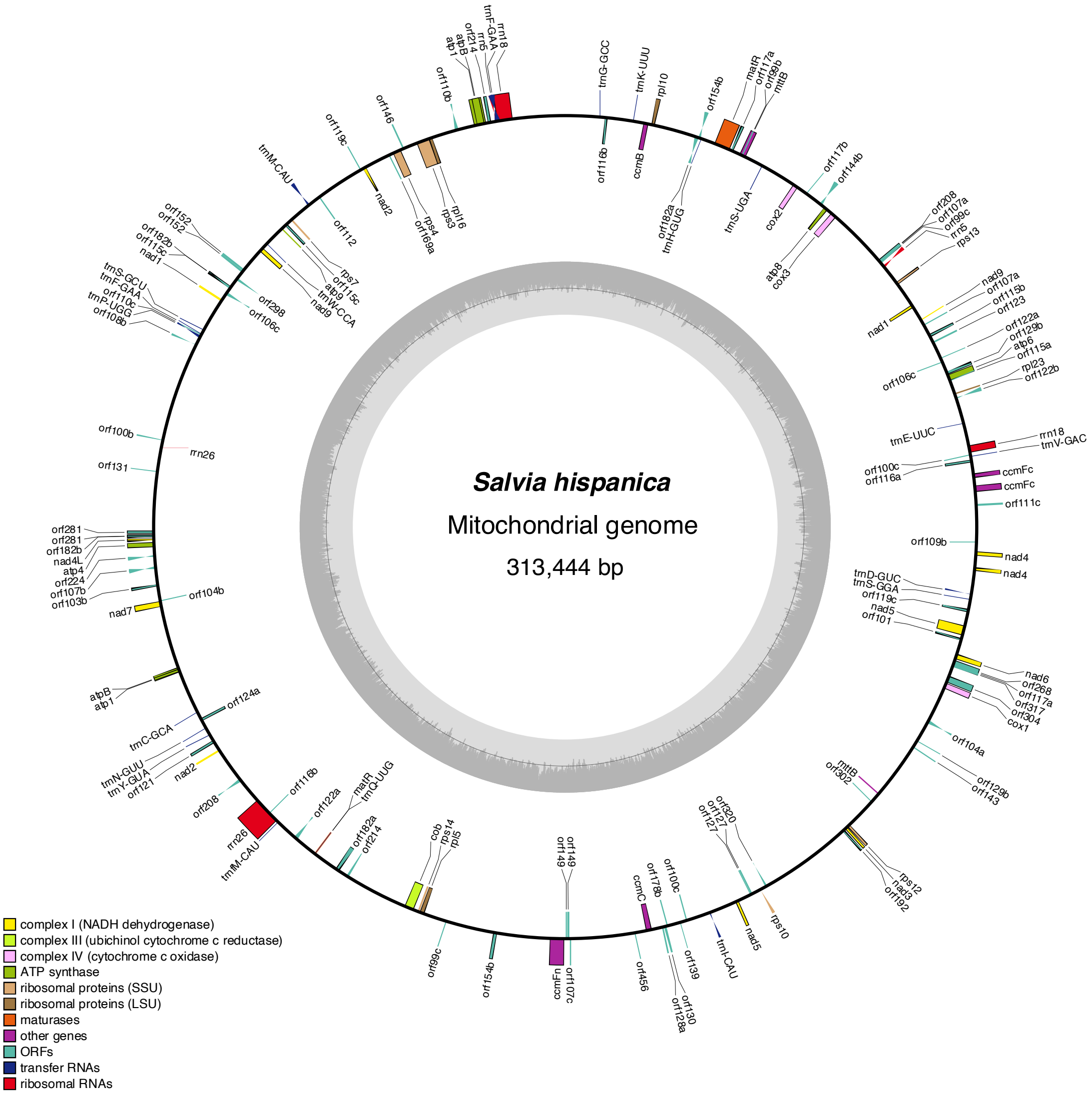

Supplemental Figure 4. Chia mitochondrial genome.

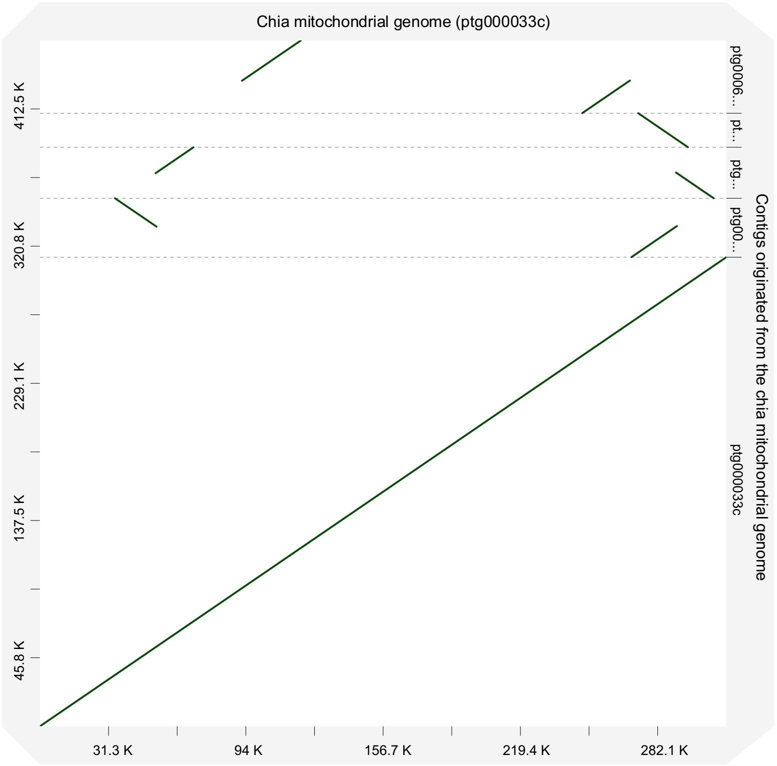

Supplemental Figure 5. Alignment of five mitochondrion-originated contigs against chia mitochondrial genome.

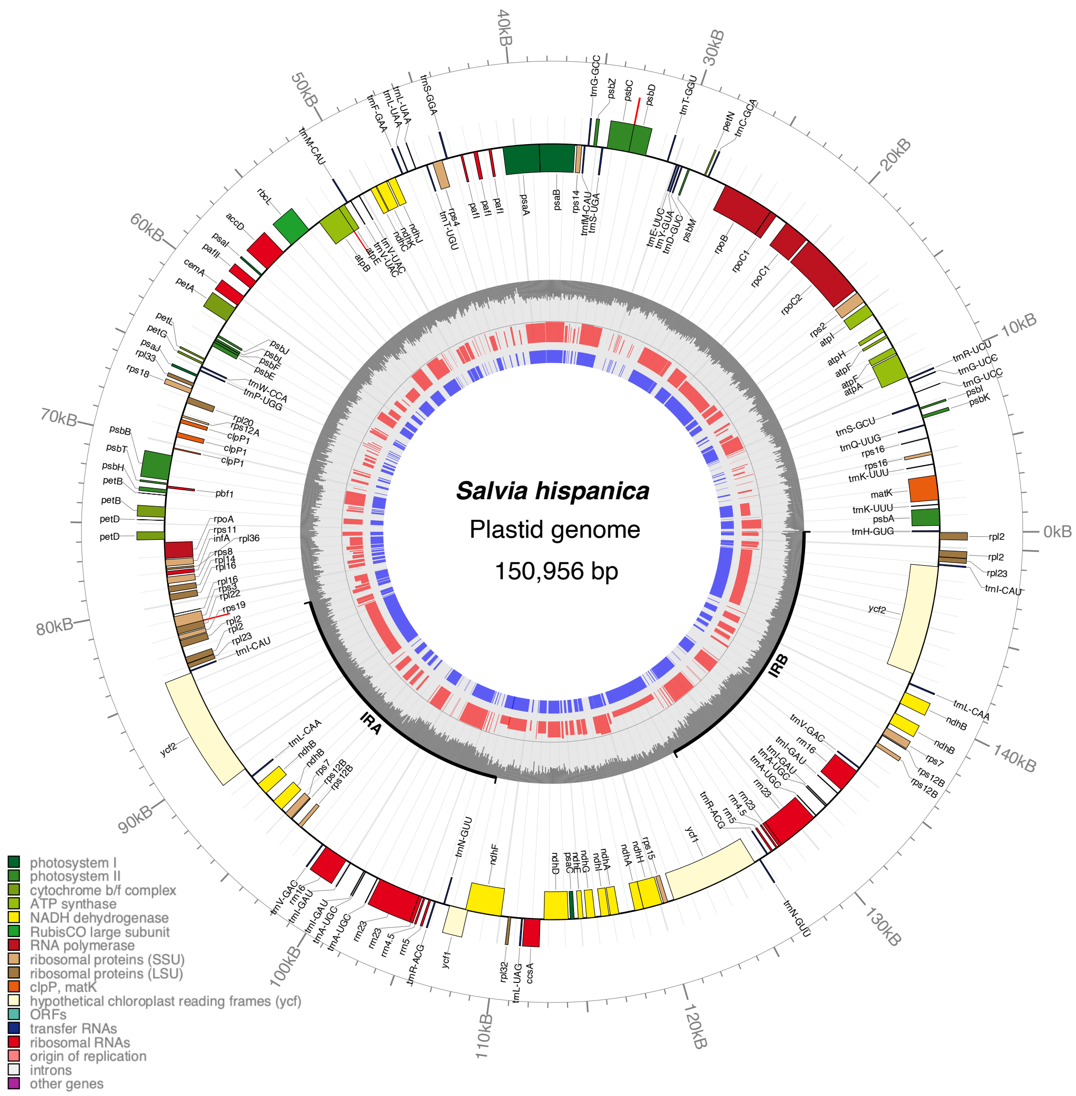

Supplemental Figure 6. Chia plastid genome

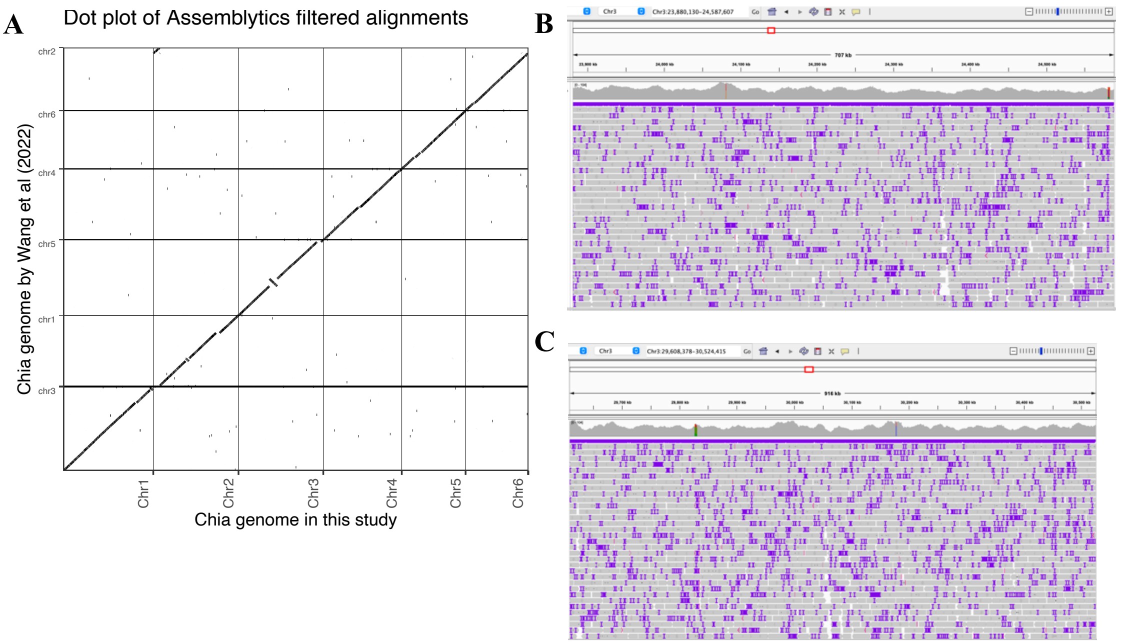

Supplemental Figure 7. A) Dot plot of synteny relationship between the chia genome in this study and one recently published chia genome by Wang et al (2022). The two boundaries of the rearrangement in Chr3 in this study was examined by visualizing HiFi reads alignment through IGV. The position of the left boundary was Chr3:24093160 (**B**) and the position of the right boundary was Chr3:30158304 (**C**).

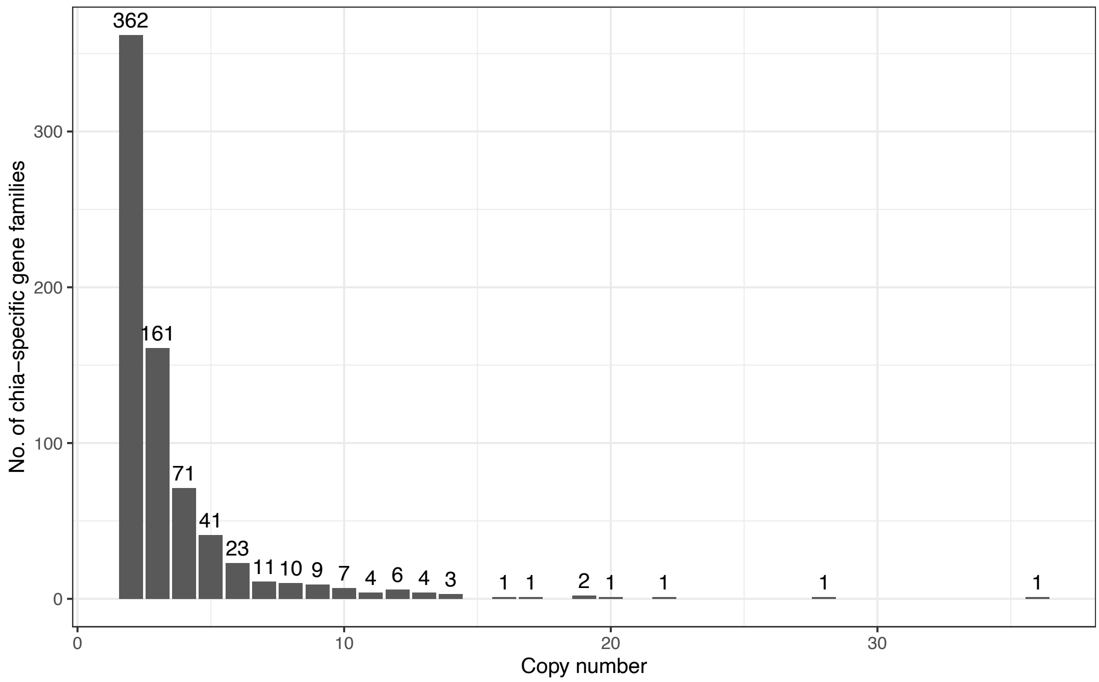

Supplemental Figure 8. Copy number distribution of chia-specific gene families.

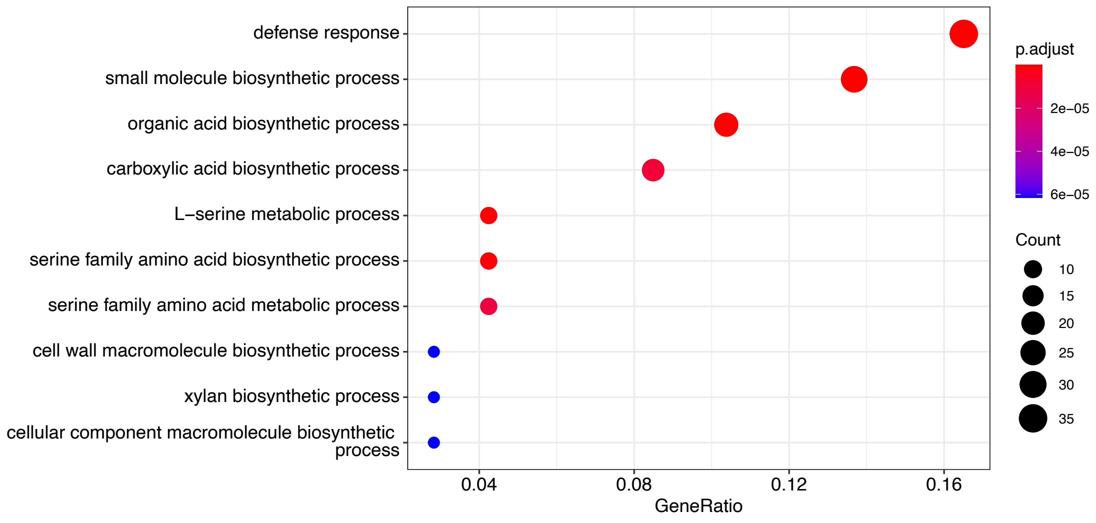

Supplemental Figure 9. Gene Ontology enrichment of genes in the chia-specific gene families in the category of biological process (BP).

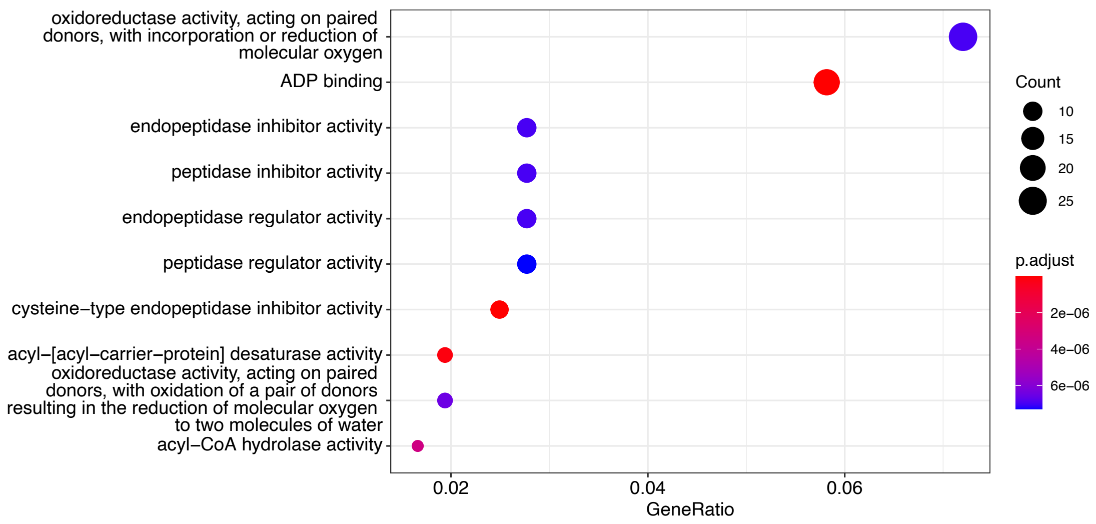

Supplemental Figure 10. Gene Ontology enrichment of genes in the chia-specific gene families in the category of molecular function (MF).

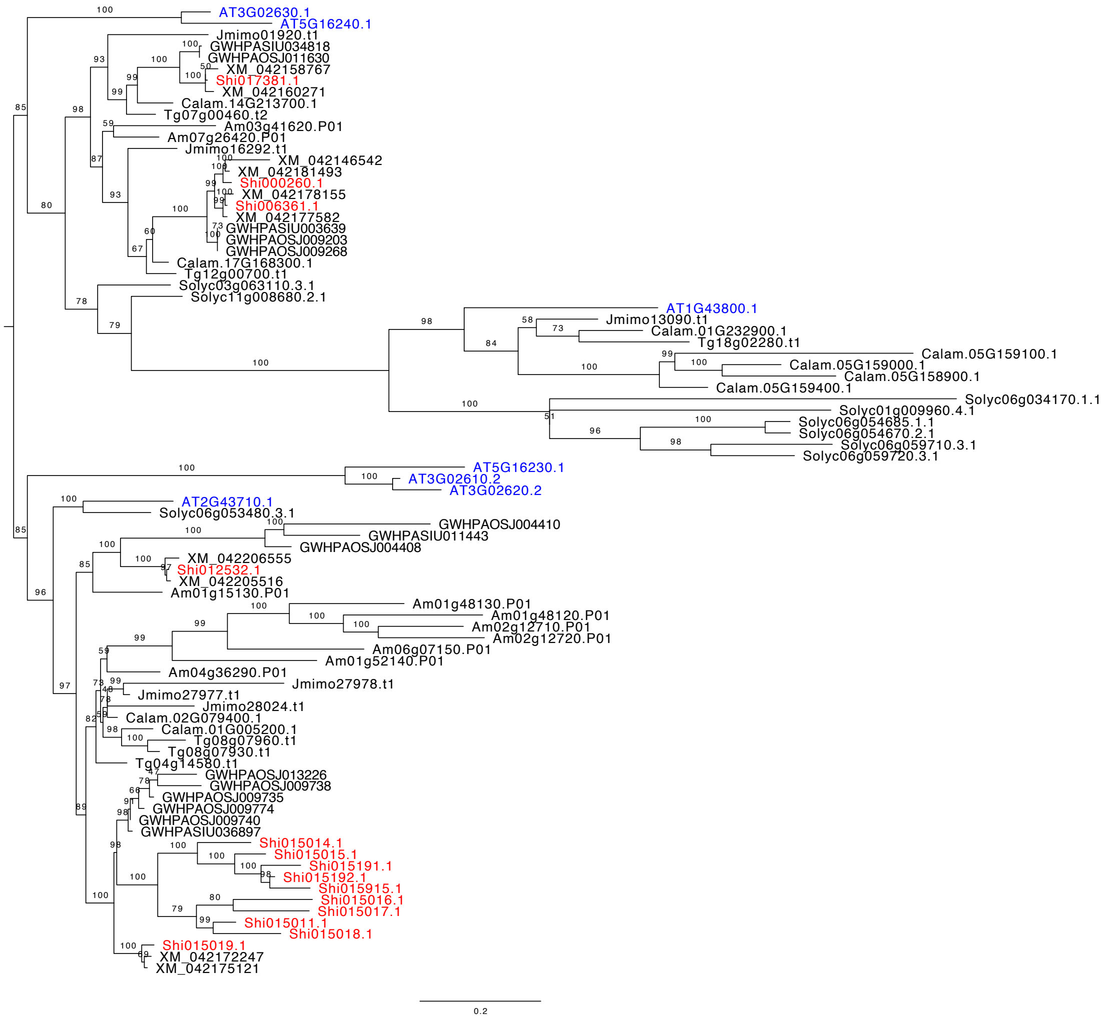

Supplemental Figure 11. Phylogenetic tree of Arabidopsis *FAB2/AAD* genes and their homologs in the chia genome and other eight genomes. Shi: *Salvia hispanica*; AT: *Arabidopsis thaliana*; XM: *Salvia splendens*; GWHTAOSJ: *Salvia miltiorrhiza*; GWHTASIU: *Salvia bowleyana*; Tg: *Tectona grandis*; Jmimo: *Jacaranda mimosifolia*; Calam: *Callicarpa americana*; Am: *Antirrhinum majus*; Solyc: *Solanum lycopersicum*.

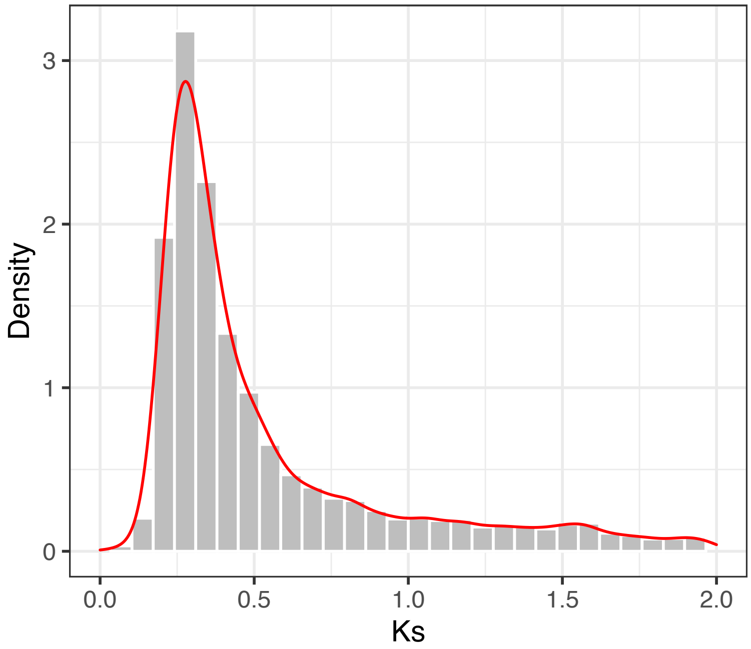

Supplemental Figure 12. Ks histogram and density plot of chia paralogs.

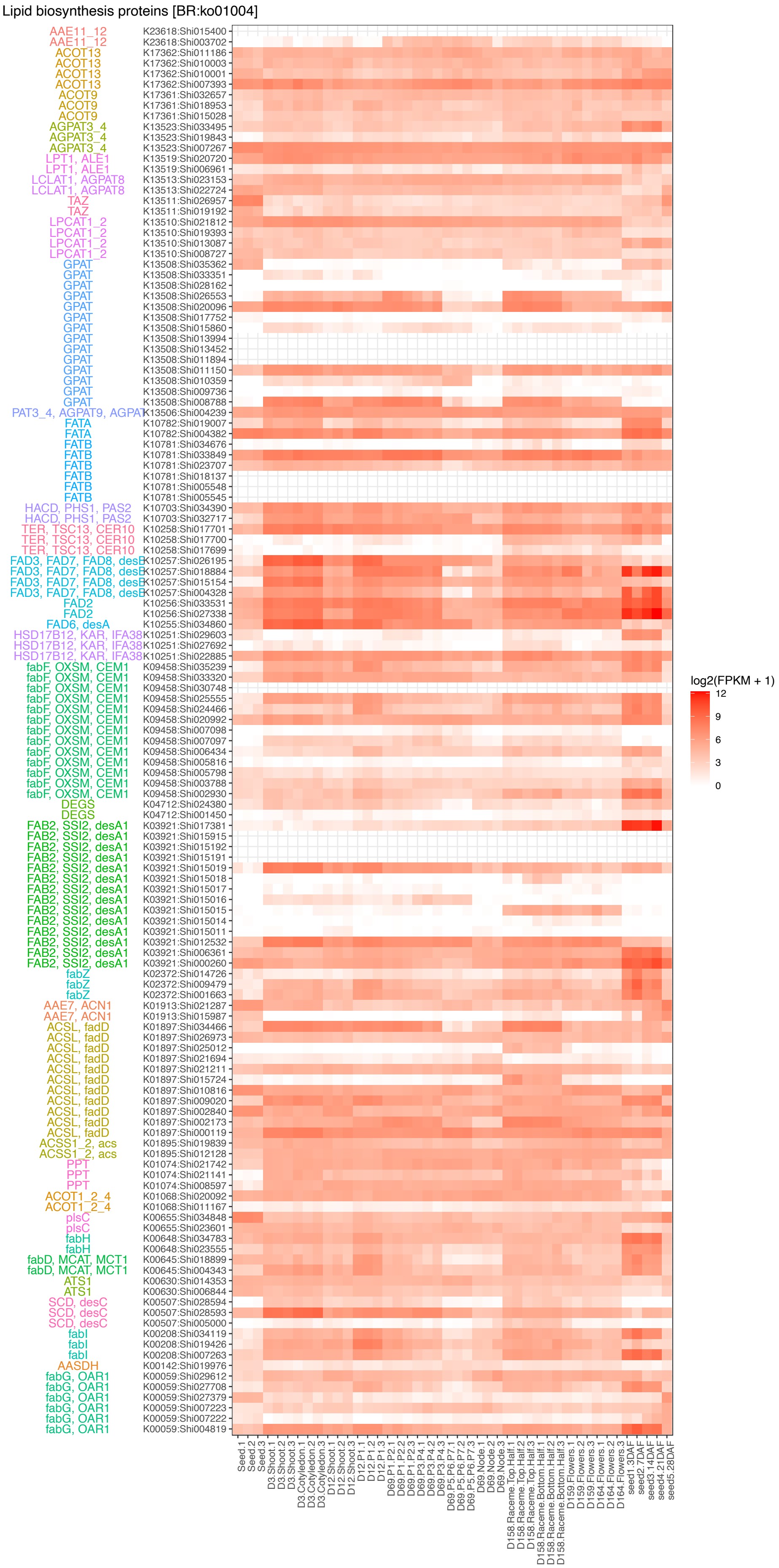
Lipid biosynthesis proteins [BR:ko01004]
AAE11_12 K23618:Shi015400
AAE11_12 K23618:Shi003702
ACOT13 K17362:Shi011186
ACOT13 K17362:Shi010003
ACOT13 K17362:Shi010001
ACOT13 K17362:Shi007393
ACOT9 K17361:Shi032657
ACOT9 K17361:Shi018953
ACOT9 K17361:Shi015028
AGPAT3_4 K13523:Shi033495
AGPAT3_4 K13523:Shi019843
AGPAT3_4 K13523:Shi007267
LPT1, ALE1 K13519:Shi020720
LPT1, ALE1 K13519:Shi006961
LCLAT1, AGPAT8 K13513:Shi023153
LCLAT1, AGPAT8 K13513:Shi022724
TAZ K13511:Shi026957
TAZ K13511:Shi019192
LPCAT1_2 K13510:Shi021812
LPCAT1_2 K13510:Shi019393
LPCAT1_2 K13510:Shi013087
LPCAT1_2 K13510:Shi008727
GPAT K13508:Shi035362
GPAT K13508:Shi033351
GPAT K13508:Shi028162
GPAT K13508:Shi026553
GPAT K13508:Shi020096
GPAT K13508:Shi017752
GPAT K13508:Shi015860
GPAT K13508:Shi013994
GPAT K13508:Shi013452
GPAT K13508:Shi011894
GPAT K13508:Shi011150
GPAT K13508:Shi010359
GPAT K13508:Shi009736
GPAT K13508:Shi008788
PAT3_4, AGPAT9, AGPAT K13506:Shi004239
FATA K10782:Shi019007
FATA K10782:Shi004382
FATB K10781:Shi034676
FATB K10781:Shi033849
FATB K10781:Shi023707
FATB K10781:Shi018137
FATB K10781:Shi005548
FATB K10781:Shi005545
HACD, PHS1, PAS2 K10703:Shi034390
HACD, PHS1, PAS2 K10703:Shi032717
TER, TSC13, CER10 K10258:Shi017701
TER, TSC13, CER10 K10258:Shi017700
TER, TSC13, CER10 K10258:Shi017699
FAD3, FAD7, FAD8, desB K10257:Shi026195
FAD3, FAD7, FAD8, desB K10257:Shi018884
FAD3, FAD7, FAD8, desB K10257:Shi015154
FAD3, FAD7, FAD8, desB K10257:Shi004328
FAD2 K10256:Shi033531
FAD2 K10256:Shi027338
FAD6, desA K10255:Shi034860
HSD17B12, KAR, IFA38 K10251:Shi029603
HSD17B12, KAR, IFA38 K10251:Shi027692
HSD17B12, KAR, IFA38 K10251:Shi022885
fabF, OXSM, CEM1 K09458:Shi035239
fabF, OXSM, CEM1 K09458:Shi033320
fabF, OXSM, CEM1 K09458:Shi030748
fabF, OXSM, CEM1 K09458:Shi025555
fabF, OXSM, CEM1 K09458:Shi024466
fabF, OXSM, CEM1 K09458:Shi020992
fabF, OXSM, CEM1 K09458:Shi007098
fabF, OXSM, CEM1 K09458:Shi007097
fabF, OXSM, CEM1 K09458:Shi006434
fabF, OXSM, CEM1 K09458:Shi005816
fabF, OXSM, CEM1 K09458:Shi005798
fabF, OXSM, CEM1 K09458:Shi003788
fabF, OXSM, CEM1 K09458:Shi002930
DEGS K04712:Shi024380
DEGS K04712:Shi001450
FAB2, SSI2, desA1 K03921:Shi017381
FAB2, SSI2, desA1 K03921:Shi015915
FAB2, SSI2, desA1 K03921:Shi015192
FAB2, SSI2, desA1 K03921:Shi015191
FAB2, SSI2, desA1 K03921:Shi015019
FAB2, SSI2, desA1 K03921:Shi015018
FAB2, SSI2, desA1 K03921:Shi015017
FAB2, SSI2, desA1 K03921:Shi015016
FAB2, SSI2, desA1 K03921:Shi015015
FAB2, SSI2, desA1 K03921:Shi015014
FAB2, SSI2, desA1 K03921:Shi015011
FAB2, SSI2, desA1 K03921:Shi012532
FAB2, SSI2, desA1 K03921:Shi006361
FAB2, SSI2, desA1 K03921:Shi000260
fabZ K02372:Shi014726
fabZ K02372:Shi009479
fabZ K02372:Shi001663
AAE7, ACN1 K01913:Shi021287
AAE7, ACN1 K01913:Shi015987
ACSL, fadD K01897:Shi034466
ACSL, fadD K01897:Shi026973
ACSL, fadD K01897:Shi025012
ACSL, fadD K01897:Shi021694
ACSL, fadD K01897:Shi021211
ACSL, fadD K01897:Shi015724
ACSL, fadD K01897:Shi010816
ACSL, fadD K01897:Shi009020
ACSL, fadD K01897:Shi002840
ACSL, fadD K01897:Shi002173
ACSL, fadD K01897:Shi000119
ACSS1_2, acs K01895:Shi019839
ACSS1_2, acs K01895:Shi012128
PPT K01074:Shi021742
PPT K01074:Shi021141
PPT K01074:Shi008597
ACOT1_2_4 K01068:Shi020092
ACOT1_2_4 K01068:Shi011167
plsC K00655:Shi034848
plsC K00655:Shi023601
fabH K00648:Shi034783
fabH K00648:Shi023555
fabD, MCAT, MCT1 K00645:Shi018899
fabD, MCAT, MCT1 K00645:Shi004343
ATS1 K00630:Shi014353
ATS1 K00630:Shi006844
SCD, desC K00507:Shi028594
SCD, desC K00507:Shi028593
SCD, desC K00507:Shi005000
fabI K00208:Shi034119
fabI K00208:Shi019426
fabI K00208:Shi007263
AASDH K00142:Shi019976
fabG, OAR1 K00059:Shi029612
fabG, OAR1 K00059:Shi027708
fabG, OAR1 K00059:Shi027379
fabG, OAR1 K00059:Shi007223
fabG, OAR1 K00059:Shi007222
fabG, OAR1 K00059:Shi004819
Seed.1
Seed.2
Seed.3
D3.Shoot.1
D3.Shoot.2
D3.Shoot.3
D3.Cotyledon.1
D3.Cotyledon.2
D3.Cotyledon.3
D12.Shoot.1
D12.Shoot.2
D12.Shoot.3
D12.P1.1
D12.P1.2
D12.P1.3
D69.P1.P2.1
D69.P1.P2.2
D69.P1.P2.3
D69.P3.P4.1
D69.P3.P4.2
D69.P3.P4.3
D69.P5.P6.P7.1
D69.P5.P6.P7.2
D69.P5.P6.P7.3
D69.Node.1
D69.Node.2
D69.Node.3
D158.Raceme.Top.Half.1
D158.Raceme.Top.Half.2
D158.Raceme.Top.Half.3
D158.Raceme.Bottom.Half.1
D158.Raceme.Bottom.Half.2
D158.Raceme.Bottom.Half.3
D159.Flowers.1
D159.Flowers.2
D159.Flowers.3
D164.Flowers.1
D164.Flowers.2
D164.Flowers.3
seed1.3DAF
seed2.7DAF
seed3.14DAF
seed4.21DAF
seed5.28DAF
log2(FPKM + 1)
12
9
6
3
0

Supplemental Figure 13. A heatmap showing the steady-state mRNA level of fatty acid biosynthesis genes in chia (in the KEGG BRITE ko01004).

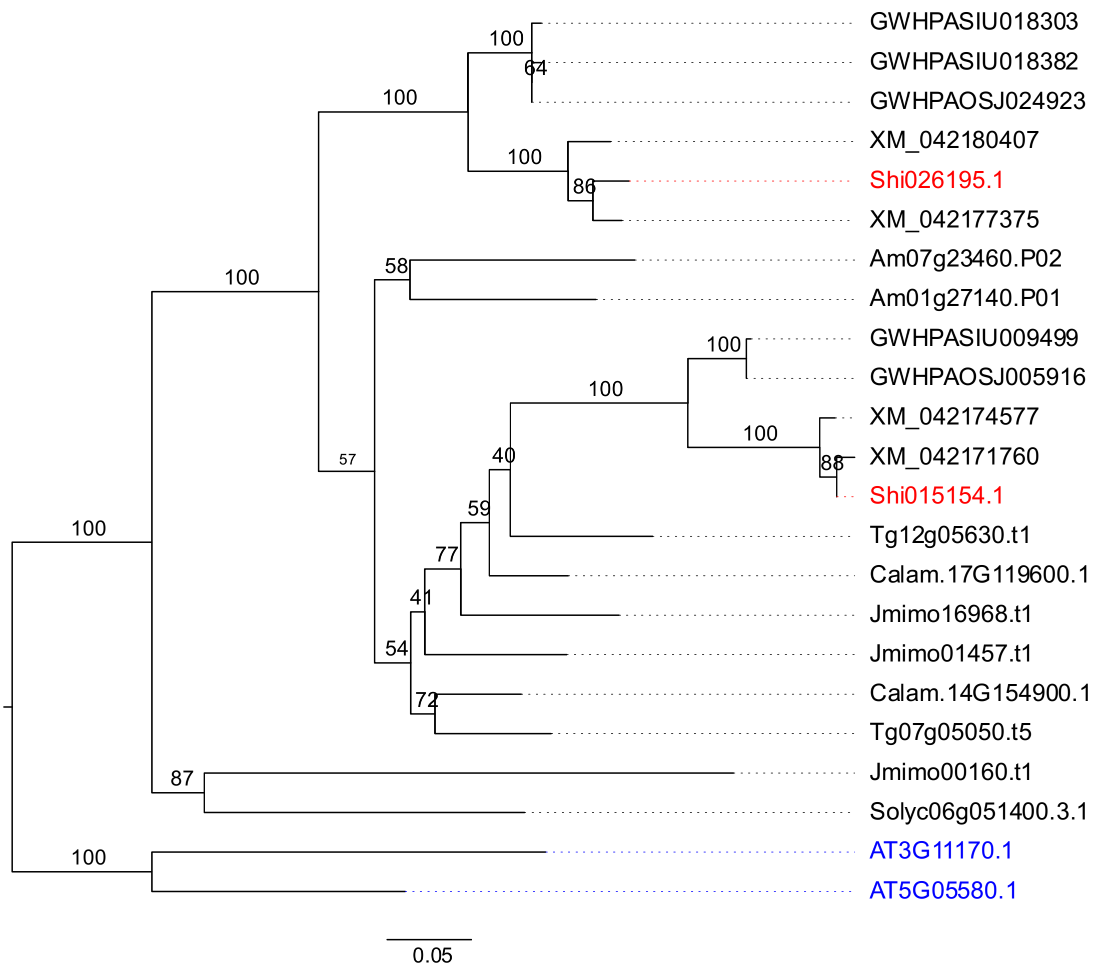

Supplemental Figure 14. Phylogenetic tree of homologous genes of Arabidopsis *FAD7* (AT3G11170) and *FAD8* (AT5G05580). Shi: *Salvia hispanica*; AT: *Arabidopsis thaliana*; XM: *Salvia splendens*; GWHTAOSJ: *Salvia miltiorrhiza*; GWHTASIU: *Salvia bowleyana*; Tg: *Tectona grandis*; Jmimo: *Jacaranda mimosifolia*; Calam: *Callicarpa americana*; Am: *Antirrhinum majus*; Solyc: *Solanum lycopersicum*.

```
        Shi015154.1   MASWVLSGCGLKPLPRIYPMPRTVSSPN-PSKLRISTADF--------------SSDSSS   45
        Shi026195.1   MASFVISGCGLKPLPRIYPKPRSVQNSFSTSNLRISRPNQ----------------FSSS   44
FAD7/8  AT3G11170.1   MANLVLSECGIRPLPRIYTTPRSNFLSNNN---KFRPSLS--SSSYKTSSSPLSFGLNSR   55
        AT5G05580.1   MASSVLSECGFRPLPRFYPKHTTSFASNPKPTFKFNPPL-----------KPPSSLLNSR   49
        AT2G29980.1   ------------------------------------------------------------   0
FAD3    Shi004328.1   ----------MRSLQSHTPTRI---CAHSLSLYIYSHSRLFHSFKLKTSSSSPK------   41
        Shi018884.1   ------------------------------------------------------------   0

        Shi015154.1   LCSVGRGRNWGLNVSAPLRFQEVG--EEENEERESEVVN--GFGGGDGFDPGAPPPFKLA   101
        Shi026195.1   SIGINQKRNWGLGVSAPLRIQPLE--EEN-----------------EEFDPAAPPPFKLS   85
FAD7/8  AT3G11170.1   DG---FTRNWALNVSTPLTTPIFE--ESPLEEDN-----------KQRFDPGAPPPFNLA   99
        AT5G05580.1   YGFYSKTRNWALNVATPLTTLQ------SPSEED-----------TERFDPGAPPPFNLA   92
        AT2G29980.1   -----------MVVAMDQRT----NVNGDPGAG--------DRKKEERFDPSAQPPFKIG   37
FAD3    Shi004328.1   -----------MAVSSGARLSESGAEGGEPYAGQCEHLEGIGKRAADKFDPAAPPPFKIA   90
        Shi018884.1   -----------MAVSSGADAEHH----------GHAQYEHLGKRAADKFDPAAPPPFKIA   39
                                 : *:                               : ***.* ***::.

        Shi015154.1   DIRAAIPKHCWVKNPWKSMSYVVRDVAVVFGLAAAAAYLNNWAVWPLYWFAQGTMFWALF   161
        Shi026195.1   DIKAAIPKHCWVKDPWRSVGYVVRDVVAVLGMAAAAAYFNSWIVWPLYWFAQSTMFWALF   145
FAD7/8  AT3G11170.1   DIRAAIPKHCWVKNPWKSLSYVVRDVAIVFALAAGAAYLNNWIVWPLYWLAQGTMFWALF   159
        AT5G05580.1   DIRAAIPKHCWVKNPWMSMSYVVRDVAIVFGLAAVAAYFNNWLLWPLYWFAQGTMFWALF   152
        AT2G29980.1   DIRAAIPKHCWVKSPLRSMSYVVRDIIAVAALAIAAVYVDSWFLWPLYWAAQGTLFWAIF   97
FAD3    Shi004328.1   DIRAAIPPHCWVKDPLRSLSYVAWDLIAVAALLAAAAYFDSWIFWPIYWAAQGTMFWALF   150
        Shi018884.1   DIRAAIPPHCWVKDPLRSLSYVAWDVFVVAALLAAAAFFDSWIFWPIYWAAQGTMFWALF   99
              **:**** *****.*  *:.**. *:  * .:   *.:.:.* .**:** **.*:***:*

        Shi015154.1   VLGHDCGHGSFSNDPKLNSVAGHLLHSSILVPYHGWRISHRTHHQNHGHVENDESWHPLS   221
        Shi026195.1   VLGHDCGHGSFSNNPKLNSVFGHFLHSSILVPYHGWRISHRTHHQNHGHVENDESWHPMP   205
FAD7/8  AT3G11170.1   VLGHDCGHGSFSNDPKLNSVVGHLLHSSILVPYHGWRISHRTHHQNHGHVENDESWHPMS   219
        AT5G05580.1   VLGHDCGHGSFSNDPRLNSVAGHLLHSSILVPYHGWRISHRTHHQNHGHVENDESWHPLP   212
        AT2G29980.1   VLGHDCGHGSFSDIPLLNSVVGHILHSFILVPYHGWRISHRTHHQNHGHVENDESWVPLP   157
FAD3    Shi004328.1   VLGHDCGHGSFSDSTTLNNVVGHILHSSILVPYHGWRISHRTHHQNHGHVEKDESWVPLP   210
        Shi018884.1   VLGHDCGHGSFSDNTTLNNVVGHVLHSSILVPYHGWRISHRTHHQNHGHVENDESWVPLT   159
              ************:   **.* **.*** ***********************:**** *:

        Shi015154.1   EKIYKQLDFVTKKLRFTLPFPMLAYPIYLWSRSPGKKGSHFHPDSDLFVPNERKDVITST   281
        Shi026195.1   EKIYNSLDSMAKKLRFTLPFPMLAYPIYLWTRSPGKKGSHYHPDSDLFVPAERKDVITST   265
FAD7/8  AT3G11170.1   EKIYNTLDKPTRFFRFTLPLVMLAYPFYLWARSPGKKGSHYHPDSDLFLPKERKDVLTST   279
        AT5G05580.1   ESIYKNLEKTTQMFRFTLPFPMLAYPFYLWNRSPGKQGSHYHPDSDLFLPKEKKDVLTST   272
        AT2G29980.1   ERVYKKLPHSTRMLRYTVPLPMLAYPLYLCYRSPGKEGSHFNPYSSLFAPSERKLIATST   217
FAD3    Shi004328.1   ENLYKQLDFSTKFLRYKIPFPMFAYPLYLWYRSPGKTGSHFNPDSSLFKPNERDLVITST   270
        Shi018884.1   ENLYKQLDFSTKFLRYKIPFPMFAYPLYLWYRSPGKSGSHFNPYSSLFKPNERDLVITST   219
              * :*: *   :: :*:.:*: *:***:**  ***** ***::* *.** * *:. : ***

        Shi015154.1   VCWTAMVAILAGLSFVMGPLQLLKLYGIPYFGFVAWLDLVTYLHHHGHEDKLPWYRGKEW   341
        Shi026195.1   VCWTAMAALLVGLSFVMGPIQLLKLYGIPYLGFVAWLDTVTYLHHHGHEDKLPWYRGKEW   325
FAD7/8  AT3G11170.1   ACWTAMAALLVCLNFTIGPIQMLKLYGIPYWINVMWLDFVTYLHHHGHEDKLPWYRGKEW   339
        AT5G05580.1   ACWTAMAALLVCLNFVMGPIQMLKLYGIPYWIFVMWLDFVTYLHHHGHEDKLPWYRGKEW   332
        AT2G29980.1   TCWSIMFVSLIALSFVFGPLAVLKVYGVPYIIFVMWLDAVTYLHHHGHDEKLPWYRGKEW   277
FAD3    Shi004328.1   VCWAAMVAFLLYASTIVGPTMLFKLYGVPYLIFVVWLDTVTYLHHHGYDKKLPWYRSKEW   330
        Shi018884.1   ICWAAMVACLLYASTIVGPTMLFKLYGVPYLIFVVWLDTVTYLHHHGYDKKLPWYRSKEW   279
               **: * . *   .  .** ::*:**:**   * *** ********::.******.***

        Shi015154.1   SYLRGGLTTLDRDYGWINNIHHDIGTHVIHHLFPQIPHYHLIEATEAAKPVLGKYYKEPQ   401
        Shi026195.1   SYLRGGLTTLDRDYGLINNIHHDIGTHVIHHLFPQIPHYNLIEATEAAKGVLGKYYREPK   385
FAD7/8  AT3G11170.1   SYLRGGLTTLDRDYGLINNIHHDIGTHVIHHLFPQIPHYHLVEATEAAKPVLGKYYREPD   399
        AT5G05580.1   SYLRGGLTTLDRDYGWINNIHHDIGTHVIHHLFPQIPHYHLVEATEAAKPVLGKYYREPK   392
        AT2G29980.1   SYLRGGLTTIDRDYGIFNNIHHDIGTHVIHHLFPQIPHYHLVDATKAAKHVLGRYYREPK   337
FAD3    Shi004328.1   SYLRGGLTTVDQDYGIFNKIHHDIGTHVIHHLFPQIPHYHLVEATREAKRVLGNYYREPR   390
        Shi018884.1   SYLRGGLTTVDQDYGIFNKIHHDIGTHVVHHLFPQIPHYHLVEATREAKRVLGNYYREPR   339
              *********:*:*** :*:*********:**********:*::**. ** ***.**:**

        Shi015154.1   KSGPLPLYLLGVLAKSMKKDHYVSDTGDIVYYQTDPKLN----------   440
        Shi026195.1   KSGPLPLHLLGDLVRSLKKDHYVSDTGDVVYYQTDPQLNGGQKS-----   429
FAD7/8  AT3G11170.1   KSGPLPLHLLEILAKSIKEDHYVSDEGEVVYYKADPNLYGEVKVRAD--   446
        AT5G05580.1   NSGPLPLHLLGSLIKSMKQDHFVSDTGDVVYYEADPKLNGQRT------   435
        AT2G29980.1   TSGAIPIHLVESLVASIKKDHYVSDTGDIVFYETDPDLYVYASDKSKIN   386
FAD3    Shi004328.1   KSGPVPFHLIPTLLKSLSRDHYVSDNGDIVYYQTDSQLFSSKEI-----   434
        Shi018884.1   KSGAVPFHLVPTLLKSLSRDHYVSDNGDIVYYQTDGELFSSKEI-----   383
              .** :*::*:  *  *:..**:***  *::*:*::* .*
```

Supplemental Figure 15. Multiple sequence alignment of Arabidopsis *FAD3* (*AT2G29980.1*), *FAD7* (*AT3G11170.1*), *FAD8* (*AT5G05580.1*) and their homologs in

the chia genome. *Shi004328.1* and *Shi018884.1* are homologs to Arabidopsis *FAD3*. *Shi015154.1* and *Shi026195.1* are homologs to Arabidopsis *FAD7/8.* The plastid transit peptides in the N-terminal were marked in the black box.

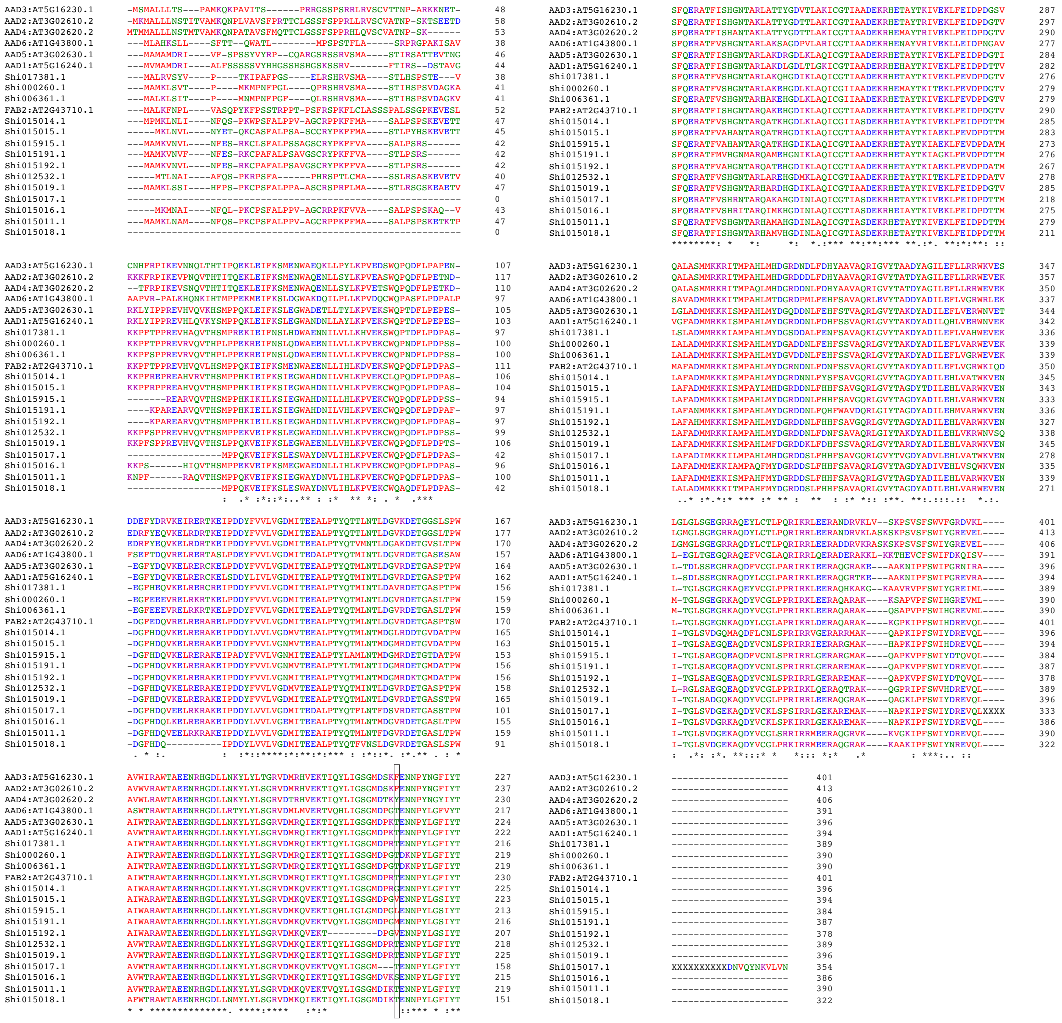

Supplemental Figure 16. Multiple sequence alignment of Arabidopsis and chia AAD/FAB2 genes. The residue that was sufficient to confer Δ9 palmitoyl-ACP desaturases (PAD) to Δ9 stearoyl-ACP desaturases (SAD) (Troncoso-Ponce *et al.*, 2016) was marked in black box in the alignment.

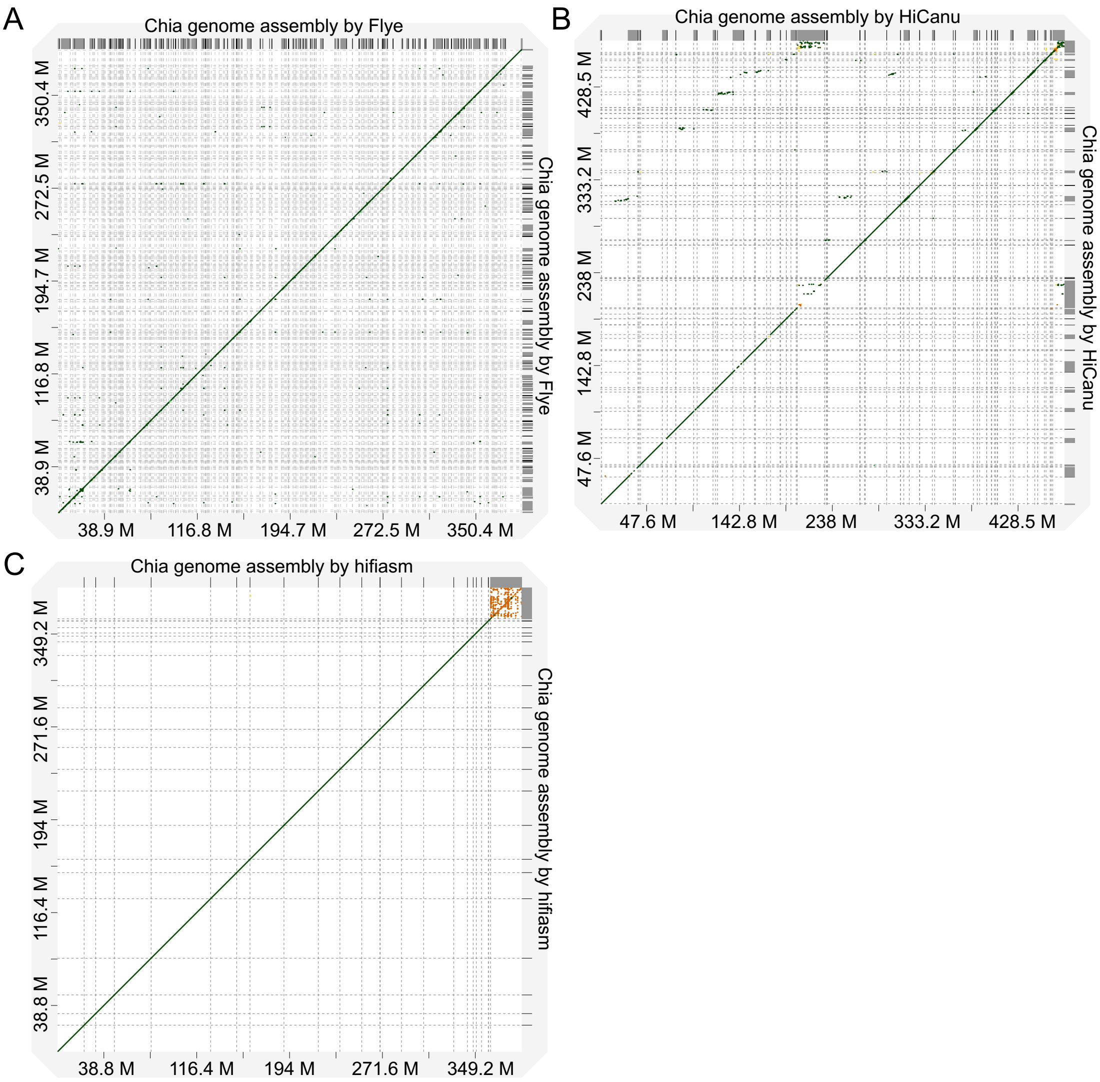

Supplemental Figure 17. Self-comparison of genome assemblies that assembled by Flye, HiCanu and hifiasm.
